# Phosphoglycerate mutase family member 5 maintains oocyte quality via mitochondrial dynamic rearrangement during aging

**DOI:** 10.1111/acel.13546

**Published:** 2022-01-07

**Authors:** Chia‐Jung Li, Li‐Te Lin, Hsiao‐Wen Tsai, Zhi‐Hong Wen, Kuan‐Hao Tsui

**Affiliations:** ^1^ Department of Obstetrics and Gynaecology Kaohsiung Veterans General Hospital Kaohsiung Taiwan; ^2^ Institute of Biopharmaceutical Sciences National Sun Yat‐sen University Kaohsiung Taiwan; ^3^ Department of Obstetrics and Gynaecology National Yang‐Ming University School of Medicine Taipei Taiwan; ^4^ Department of Marine Biotechnology and Resources National Sun Yat‐sen University Kaohsiung Taiwan; ^5^ Department of Obstetrics and Gynecology Taipei Veterans General Hospital Taipei Taiwan; ^6^ Department of Pharmacy and Master Program College of Pharmacy and Health Care Tajen University Pingtung County Taiwan; ^7^ Department of Medicine Tri‐Service General Hospital National Defense Medical Center Taipei Taiwan; ^8^ College of Health and Nursing Meiho University Pingtung Taiwan

**Keywords:** cumulus cells, granulosa cells, infertility, mitochondrial dynamics, PGAM5

## Abstract

Decline in ovarian reserve with aging is associated with reduced fertility and the development of metabolic abnormalities. Once mitochondrial homeostasis is imbalanced, it may lead to poor reproductive cell quality and aging. However, Phosphoglycerate translocase 5 (PGAM5), located in the mitochondrial membrane, is associated with necroptosis, apoptosis, and mitophagy, although the underlying mechanisms associated with ovarian aging remain unknown. Therefore, we attempted to uncover whether the high phosphoglycerate mutant enzyme family member 5 (PGAM5) expression is associated with female infertility in cumulus cells, and aims to find out the underlying mechanism of action of PGAM5. We found that PGAM5 is highly expressed and positively associated with aging, and has the potential to help maintain and regulate mitochondrial dynamics and metabolic reprogramming in aging granulosa cells, ovaries of aged female mice, and elderly patients. PGAM5 undergoes activation in the aging group and translocated to the outer membrane of mitochondria, co‐regulating DRP1; thereby increasing mitochondrial fission. A significant reduction in the quality of mitochondria in the aging group, a serious imbalance, and a significant reduction in energy, causing metabolism shift toward glycolysis, were also reported. Since PGAM5 is eliminated, the mitochondrial function and metabolism of aging cells are partially reversed. A total of 70 patients undergoing in vitro fertilization (IVF) treatment were recruited in this clinical study. The high expression of PGAM5 in the cumulus cells is negatively correlated with the pregnancy rate of infertile patients. Hence, PGAM5 has immense potential to be used as a diagnostic marker.

## Graphical abstract

Li et al. found that Phosphoglycerate mutase family member 5 (PGAM5) levels are increased during female reproductive system aging and are accompanied by mitochondrial division. Overexpression of PGAM5 diminished age‐related fertility, providing evidence for a pathogenic effect of increased PGAM5. This study reveals a previously unrecognized molecular mechanism of age‐related infertility.

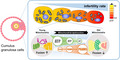

**Abbreviations**
ANOVA: analysis of variance
CC: cumulus cell
GC: granulosa cell
MMP: mitochondrial membrane potential
OGE: overlap gene
PGAM5: phosphoglycerate mutant enzyme family member 5
ROS: reactive oxygen species
RT: room temperature
SA‐β‐gal: senescence‐associated β‐galactosidase
SEM: standard error of mean

## 1 INTRODUCTION

The decline in female fertility due to ovarian aging is characterized by changes in the quantity and quality of ovarian oocyte reserves, over time (te Velde & Pearson, 2002). Oocytes contain mitochondria, which are the source of energy for the germination of immature oocytes. The maternal age is related to the increase in oxidative stress in the oocytes and lead to mitochondrial dysfunction. The oxidative damage, deletions or point mutations, and mutations in the mitochondrial genome lead to dysfunction resulting in the insufficiency of ATP and other key mitochondrial functions essential for fertilization (Agarwal et al., 2005). Oocyte senescence is due to the general dysfunction of many cellular processes that leads to cell aging. Senescence is related to the decline of the mitochondrial function, particularly in the non‐replicating cells (such as mature oocytes), responsible for the decline in female fertility (Bentov et al., 2011).

Follicles are the basic functional unit of human reproduction, and cumulus (CC) and granulosa cells (GC) play a key role in the development, maturation, and fertilization of oocytes. They manifest the regulation of oocyte meiosis and support the maturation of the oocyte cytoplasm (Huang & Wells, 2010). Additionally, they facilitate the sperm and egg to combine, and their shape and extent of expansion affect the maturity and quality of oocytes. Oocytes regulate the function of CC and GC by secreting various factors and GC from the follicular microenvironment for oocyte development (Gilchrist et al., 2008). Recent studies have focused on understanding age‐related changes in the entire ovarian microenvironment. Some of these changes occur in the granulosa cells that potentially impact the oocyte quality (Lin et al., 2017, 2017). The mitochondria in GC are essential for enabling oocytes to metabolize glucose into pyruvate, which is then transported to oocytes to produce ATP, aiding in development (Sutton‐McDowall et al., 2010). In the aged groups, there is a decrease in the mitochondrial fraction and an increase in the mitochondrial matrix density (de Bruin et al., 2004). The decline of the mitochondrial respiratory function associated with the aging of GC triggers the increased production of reactive oxygen species (ROS) (May‐Panloup et al., 2016).

The mitochondria of germ cells require extremely high energy to maintain cell physiology and a large number of mitochondrial dynamics to maintain homeostasis (Li et al., 2021). Mitochondria are highly dynamic organelles that can continuously move, fuse, and divide according to changes in the cellular energy requirements. Mitochondrial dynamics are mediated by the large dynamic GTPases (DRP1, OPA1, MFN1, and MFN2) embedded in the mitochondrial membrane. Mitochondrial fission produces new mitochondria needed for cell growth and promotes degradation and elimination of the damaged mitochondria (Twig et al., 2008). Mitochondrial fusion ensures close complementarity between organelles to meet the needs of dynamic energy. However, in the oocyte‐specific Drp1‐deficient mice, mitochondrial fission was found to maintain the ability of oocytes in an age‐dependent manner through multicellular organelle rearrangement (Udagawa et al., 2014).

The phosphoglycerate mutant enzyme family member 5 (PGAM5) is a mitochondrial protein phosphatase, which is reportedly involved in various stress responses from the mitochondrial quality control to cell death (Yu et al., 2020). The mitochondria are the largest cell energy storage, containing the OXPHOS protein that produces ATP and the β‐oxidase used as a transport fuel (Ma et al., 2020). When the human body is exposed to stress that disrupts the energy homeostasis, the mitochondria face metabolic challenges (Liu et al., 2020). PGAM5 initiates the mitochondrial fission by dephosphorylating DRP1S637 and promoting the mitochondrial translocation of DRP1 (Wang et al., 2012). Numerous studies have confirmed the involvement of PGAM5 in the development of Parkinson's disease, acute kidney injury, and hepatitis (Gu et al., 2019; Kang et al., 2015; Lu et al., 2014). However, it is unclear whether PGAM5 is involved in the regulation of aging germ cells. Presently, many studies have proposed that mitochondrial dynamics and oocytes have important functions. This study has made a novel discovery that PGAM5 is also involved in the lysis of mitochondria.

Given the importance of PGAM5 in mitochondrial dynamics, it was further investigated whether PGAM5 is related to aging and is age dependent. Through in vitro and clinical specimen analysis, it was substantiated that PGAM5 is essential for mitochondrial homeostasis. Furthermore, it was established that PGAM5 as well as DRP1 show a significant increase in the cumulus cells of the older patients experiencing infertility. Thus, PGAM5 and DRP1 together lead to cellular senescence and a decrease in ATP production, associated with aging.

## 2 RESULTS

### 2.1 Knockdown of PGAM5 prevented impairment of mitochondrial dysfunction in GC during aging

**FIGURE 1:**
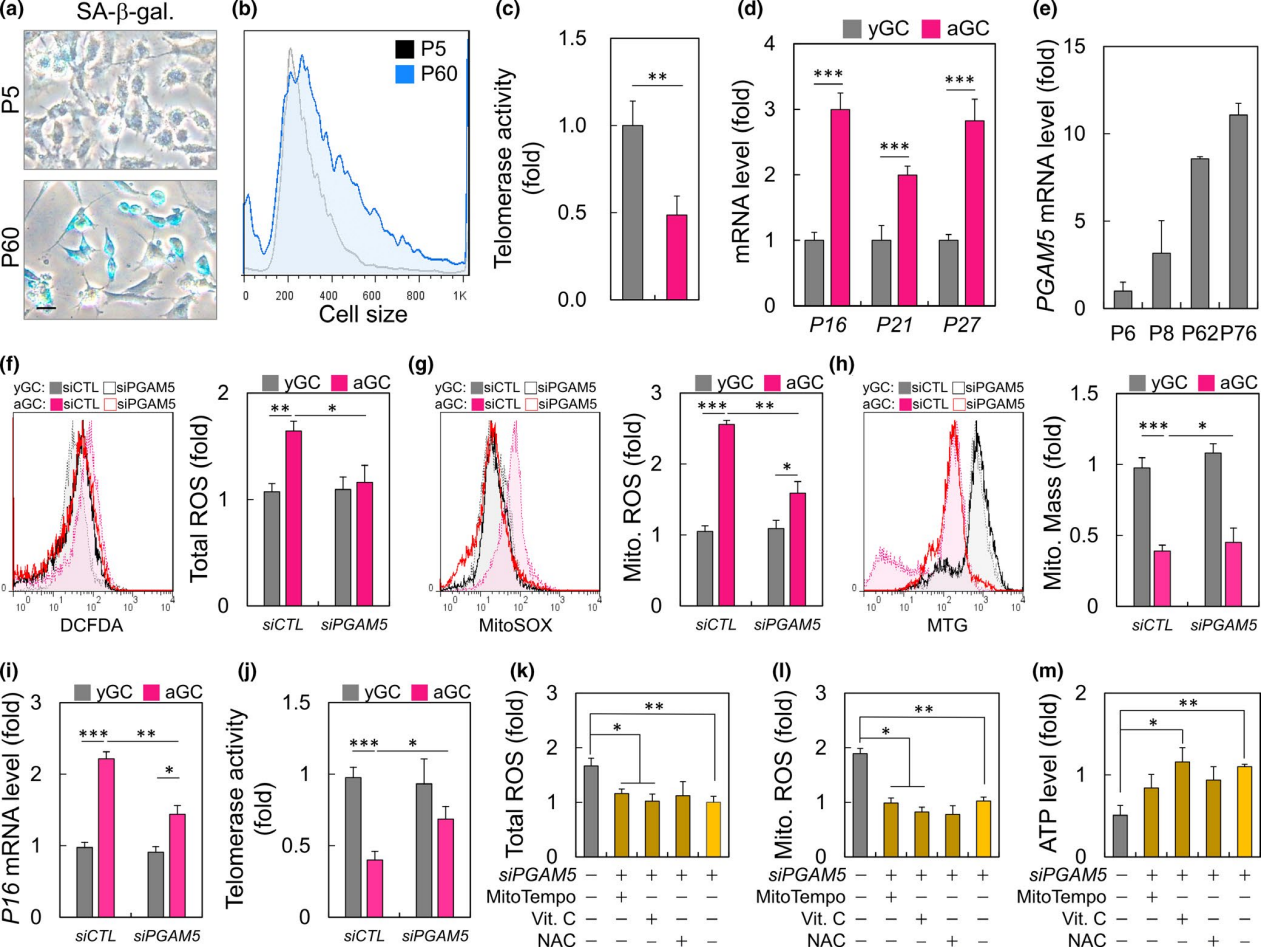
PGAM5 upregulation promotes mitochondrial dysfunction in aged GC. (a) the representative GC with senescence‐associated β‐galactosidase (SA‐β‐gal) staining from the early passage and late passage groups were shown. (b) The cells were analyzed for cell size during aging. (c) Determination of telomerase activity by qPCR. (d) The expression of aging markers in early passage (yGC) and late cells (aGC). (e) The PGAM5 expression in different GC passage. Measurement of the (f) cellular, (g) mitochondrial ROS, and (h) mitochondrial mass in GC treated with PGAM5 siRNA. (i, j) Analysis of aging marker and telomerase level in PGAM5 knockdown cells. Measurement of the (k) cellular, and (l) mitochondrial ROS in knockdown GC treated with ROS inhibitor. (m) Cellular ATP production measurements in different cell passage treated with ROS inhibitor. Scare bar =20 µm. *p < 0.05, **p < 0.01, and ***p < 0.001

To evaluate the aged granular cells (HGL5) in vitro, young granulosa cells (yGC) of early passage (<P10) and aging granulosa cells (aGC) of late passage (>P60) were established. The aging phenotype was assessed by the SA‐β‐gal activity, cell size, and telomerase activity. The β‐galactosidase activity of the older group increased, and the cell size distribution had a larger cell volume with a decrease in the telomerase activity (Figure 1a–c). To further validate the model of cellular aging, the mRNA expression for the senescence biomarkers, P16, P21, and P27 were determined to significantly increase with aging (Figure 1d). What is the biologic function within cells of increased PGAM5 in aged GC? To test whether PGAM5 participates in the aged GC, we measured *PGAM5* mRNA levels in GC and ascertained that they increased age dependently (Figure 1e). The total cell and mitochondrial ROS analysis showed that *siPGAM5* lead to a significant decrease in oxidative stress (Figure 1f–g). Interestingly, the total mass of mitochondria in aged cells did not change between the *siPGAM5* and *siCTL* groups (Figure 1h). *PGAM5* knockdown attenuated the aging progression of aGC cells, as evidenced by a decreased in P16 expression and an increased in telomerase activity (Figure 1i–j). In addition, supplementation with ROS inhibitors did not further reduce ROS (Figure 1k–l). In ATP production, Vic C and *siPGAM5* KD have the effect of adding ATP (Figure 1m).

### 2.2 The comprehensive analysis of screens reveals the differential dependence on mitochondrial dynamics signaling

**FIGURE 2:**
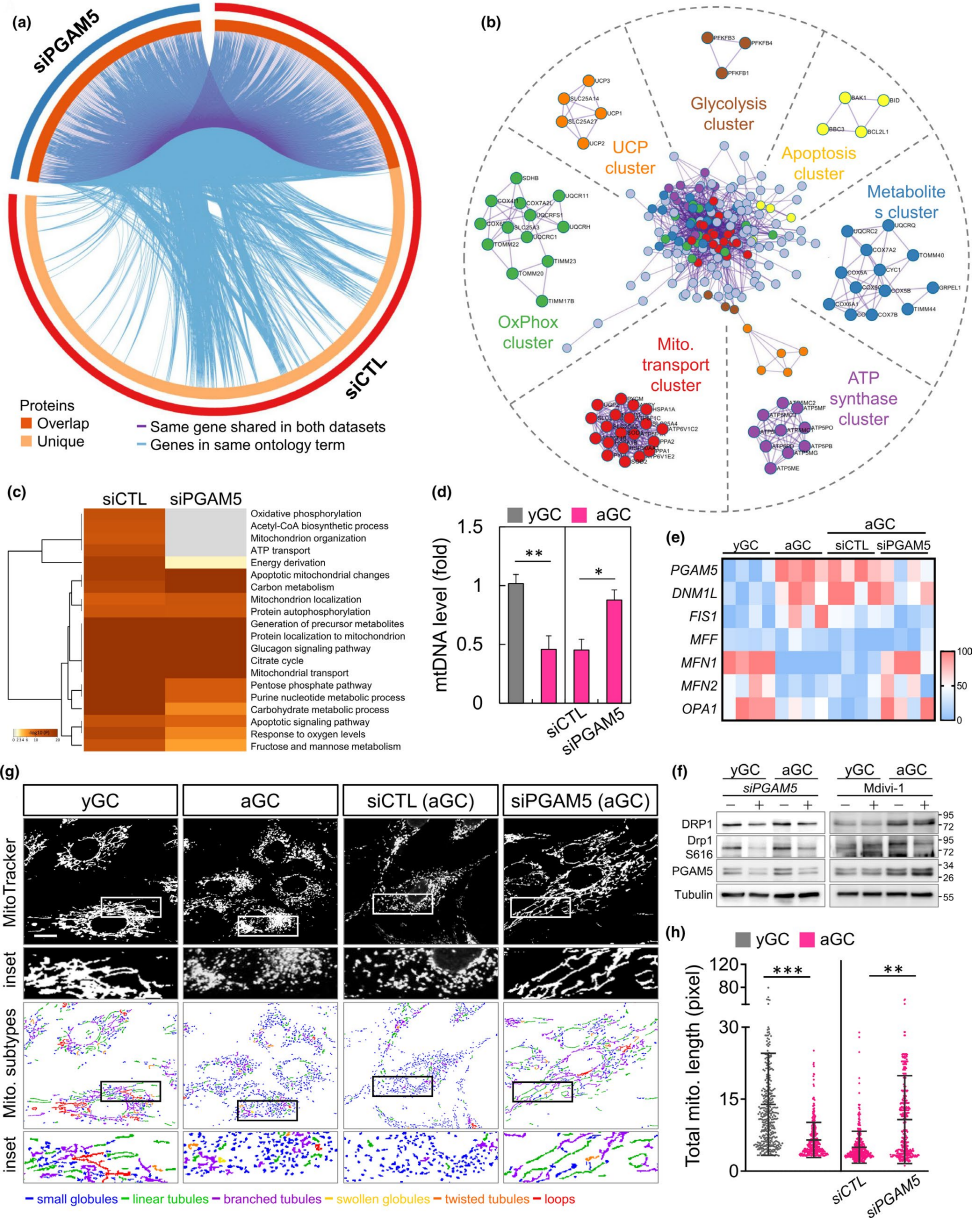
Mitochondrial dynamics and function change upon PGAM5 knockdown. (a) Pathway analysis for deregulated genes in siCTL versus siPGAM5 with interaction modules clustered based on functional similarities. MCODE algorithm was then applied to this network to identify neighborhoods where proteins are densely connected. Purple lines link the common genes among the two samples. Light blue lines link the different genes where they fall into the same ontology term. (b) Network layout of gene interactions wherein each gene is a circle node. The node size is proportional to the number of input genes that fall in that gene and color is the enrichment identity, as per the color insert at the bottom of the network. Each MCODE network is assigned a unique color. (c) Pathway enrichment of genes scoring in two or more screens within the MSigDB canonical pathways database. Top 20 enriched canonical pathways among cell lines screened. (d) Mitochondrial DNA level and (e) mitochondrial dynamics change as measured in yGC and aGC of siCTL and siPGAM5 GC. (f) Immunoblot analysis of protein expression for gene silencing and mitochondrial division inhibitor (Mdivi‐1: 20 μM). (g) Mitochondria in HGL5 cells were labeled with MitoTracker and subjected to image capture. The mitochondria were classified according to their morphology: globules, linear tubules, branched tubules, twisted tubules, and loops. (h) The total length of each mitochondrion was evaluated. Scare bar = 20 µm. *p < 0.05, **p < 0.01, and ***p < 0.001

To further gain a more in‐depth comprehension of the cellular behaviors of the overlap genes (OGEs), the functional enrichment analysis was performed in *PGAM5* KD. Figure 2a shows the overlap between the gene lists at the gene level. The gene ontology analysis revealed that the OGEs were significantly related to several mitochondrial functions, including regulation of apoptosis, OxPhos, UCP, Metabolites, ATPase, and glycolysis (Figure 2b). The cellular component analysis showed that the OGEs were mainly enriched in OxPhos, mitochondrial organization, and ATP transport. The molecular function analysis suggested that the OGEs were significantly associated with cellular response to stress, regulation of pH, and energy metabolism (Figure 2c). We further tested that the mtDNA copy number of *PGAM5* KD cells was significantly higher than that of *siCTL* KD aging cells (Figure 2d), and its effect reflected the level of genes related to mitochondrial dynamics (Figure 2e). We further validated the protein level of *PGAM5* KD and obtained consistent results with mRNA. We also tested the effect of mitochondrial fission inhibitor (Mdivi‐1) on *PGAM5* and showed that Mdivi1 not only effectively reduced the expression of DRP1‐S616 in aGC but also contributed to the compensatory increase of PGAM5 expression (Figure 2f). As shown in Figure 2g, aging causes mitochondrial fragmentation, which is indicated by the division of organelles into globular or short rods. This effect is counteracted by the depletion of *PGAM5*, which clearly limits the mitochondrial fragmentation caused by aging. Also, the average length of mitochondria was significantly higher in *siPGAM5* aGC than those in *siCTL* aGC (Figure 2h).

### 2.3 An elevated PGAM5 level and its co‐factor DRP1 in aged GC

**FIGURE 3:**
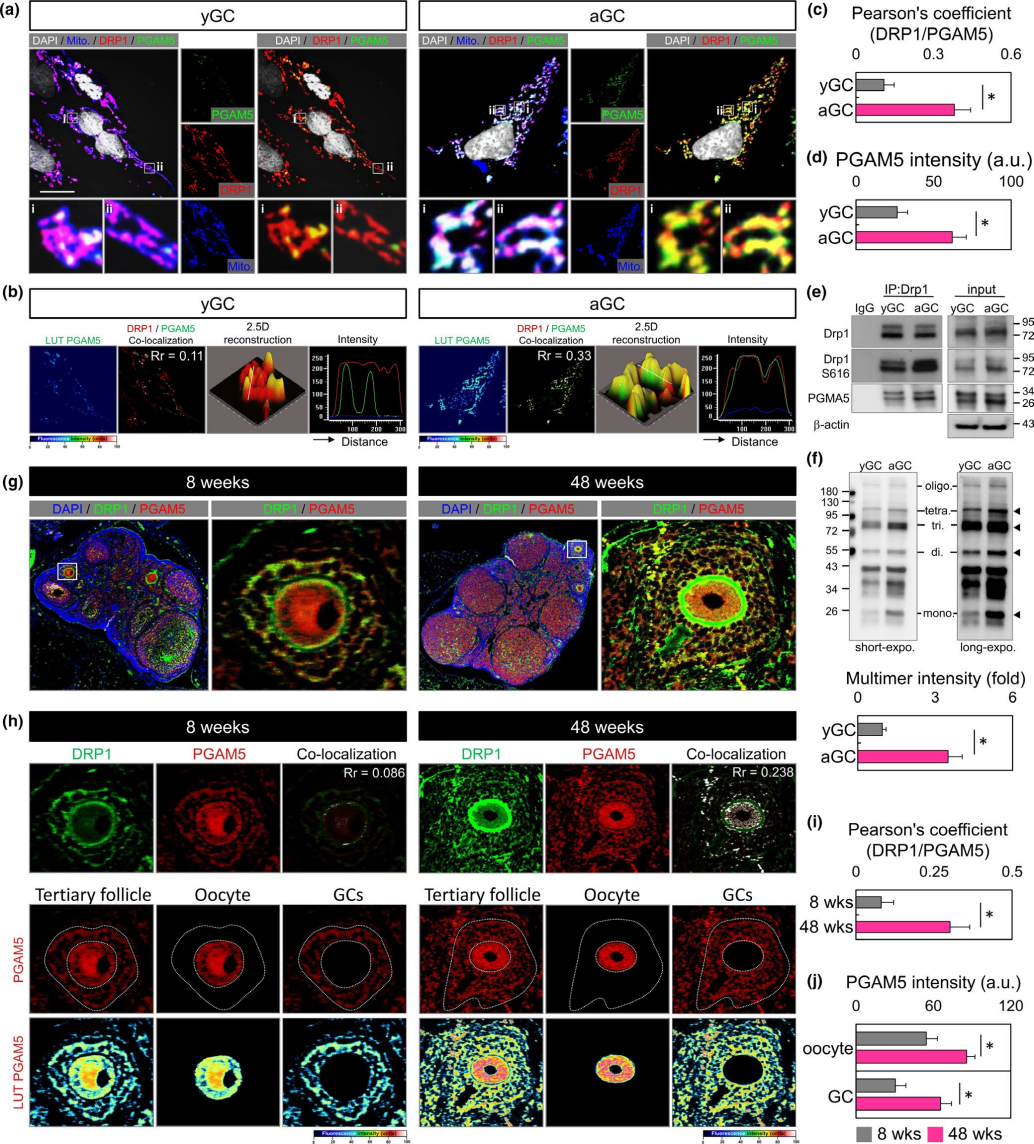
Drp1 interacts with and recruits PGAM5 to mitochondria. (a) Fluorescence images of GC stained with MitoTracker, Drp1, PGAM5, and DAPI. The enlarged images in (i, ii) highlight the representative co‐localization from white squares in the overlay images. The pseudo‐color image shows the PGAM5 fluorescence intensity. (b) PGAM5 green fluorescence is converted to a pseudocolor with a relative intensity scale. The co‐localization of DRP1 and PGAM5 are presented as the product of the differences from the mean (PDM) image. White color pixels indicate the co‐localization coefficient. A 2.5‐dimensional reconstruction and fluorescence intensity of the respective insets in (i) compared between green and red fluorescence. (c–d) Quantify (a–b) Pearson's coefficient of the colocalization and PGAM5 fluorescence intensity in yGC and aGC. (e) Immunoblot analysis of proteins immunoprecipitated with anti‐Drp1 and IgG antibodies in GC. (f) Non‐reducing SDS/PAGE immunoblot of PGAM5. Long exposure revealed tetramer and oligomer. (g) Fluorescent ICC showing expression and localization of PGAM5 and DRP1 in the ovary of mice at 8 and 48 weeks. (h) The co‐localization of DRP1 and PGAM5 is presented as the product of the differences from the mean (PDM) image; compared between green and red fluorescence. The fluorescence intensity of PGAM5 in oocytes and GC in the ovary of different ages. (i–j) Quantify (g–h) Pearson's coefficient of the colocalization and PGAM5 fluorescence intensity at different mouse age. Scare bar = 20 µm. *p < 0.05

To confirm that the abnormal mitochondrial dynamics of aGC are associated with DRP1 recruiting PGAM5, the co‐localization of the two proteins in the mitochondria of aGC was investigated. The whole‐CC were quadruple‐labeled by immunofluorescence and labeled with the PGAM5, DRP1, mitochondria, and DAPI. Similar to the previous results, the fluorescence intensity of PGAM5 increased significantly with aGC (Figure 3a). In Figure 3b, the green fluorescent PGAM5 and red fluorescent DRP1 were indicated, while two fluorescent overlaps appeared yellow, indicating that PGAM5 co‐localized with DRP1. Furthermore, the overall co‐localization inside the GC was computed and the Pearson's coefficient of the aGC was observed to significantly increase from 0.11 to 0.33 compared to the yGC (Figure 3c). The fluorescence intensity of PGAM5 was also similar to the results in Figure 1e, and aGC was significantly higher than yGC (Figure 3d). Since PGAM5 has been reported to function as a co‐factor for mitochondrial fission (Chen et al., 2020; Yu et al., 2020), it was further tested whether PGAM5 interacted properly with components of fission in aGC. Immunoprecipitation of DRP1 pulled down PGAM5 in aGC, whereas more PGAM5 were precipitated in the aGC mitochondria (Figure 3e). The non‐reducing SDS/PAGE showed that PGAM5 formed a complex with multimer in aGC. Consistently, quantification showed a defect in formation of PGAM5 oligomer in yGC (Figure 3f).

Next, we further investigated the levels of PGAM5 in the follicles of aging mice. The expression of DRP1 and PGAM5 at different ages was analyzed by scanning the ovaries with panoramic images (Figure 3g). In Figure 3h, the co‐localization coefficient of DRP1 and PGAM5 ranged from 0.086 to 0.238 at 4 and 48 weeks. The quantitative results were consistent with the co‐localization of HGL5 cells in vitro (Figure 3i). In the lower panel of 3H, we separated tertiary follicles from oocytes and GC by image analysis and quantified its fluorescence intensity individually. Our results indicated that the PGAM5 levels in the aging group were significantly higher in GC than in the young group in both in vitro and in vivo model. Consistently, high PGAM5 expression was also found in aging oocytes (Figure 3j).

### 2.4 Loss of PGAM5 promotes metabolic shift in aGC

**FIGURE 4:**
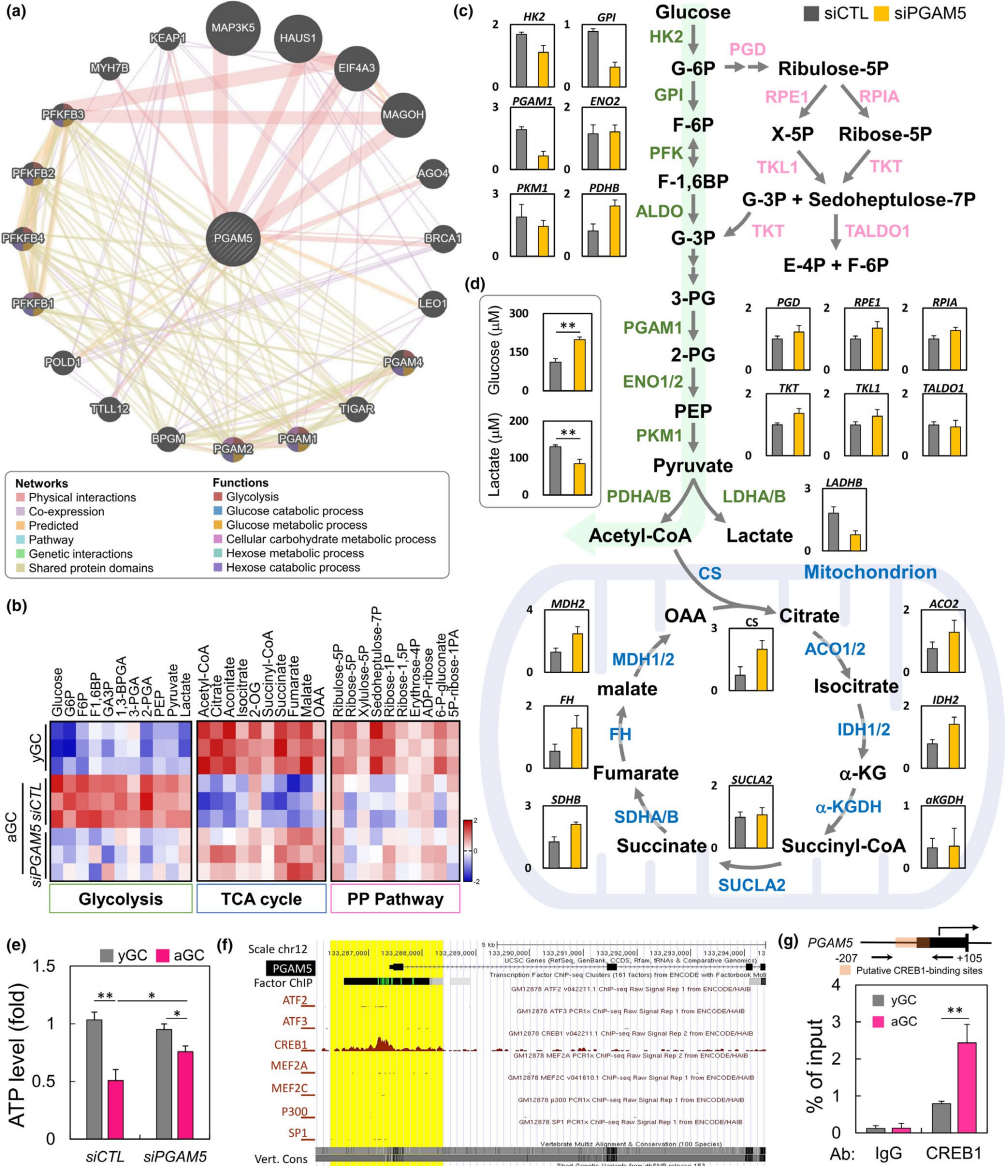
A metabolic shift from glycolysis in siCTL to OXPHOS in *siPGAM5* GC. (a) Genes associated with PGAM5 was represented by circles. The pattern of correlations between PGAM5 was stored by the GeneMANIA network. The interaction types were exhibited as indicated in the networking legend. (b) The heatmap showing metabolite levels of glycolysis, TCA cycle, and pentose phosphate pathway, based on UHPLC analysis. (c) The diagram shows the levels of enzymes metabolizing enzymes in glycolysis and TCA cycles by qPCR analysis. (d) Glucose and lactate production were elevated in siCTL and *siPGAM5* GC. (e) Cellular ATP level as measured in siCTL and *siPGAM5* GC. (f) Scheme (UCSC Genome Browser hg19) of the human genomic region encompassing the promoter of PGAM5. The brown peaks and yellow region represent the regions of CREB1 binding, according to ENCODE. (g) Chromatin immunoprecipitation assay in GC in early and late passage. Sequences containing the potential CREB1‐binding sites in *PGAM5* loci were amplified by qPCR. The upper panel shows location of primers flanking putative CREB1‐binding sites in *PGAM5* promoter region. *p < 0.05, and **p < 0.01

To further explore the role of PGAM5 and energy metabolism, the correlation between PGAM5 and glycolytic pathways were confirmed through biological information. Among them, PGAM5 interacted with KEAP1, the biomass synthesis center, and also correlated with PFKB1 to four of the PFKB family (Figure 4a). Therefore, by Ultra‐high performance liquid chromatography (UHPLC)‐MS/MS analysis of metabolites, we observed that TCA cycle and pentose phosphate pathway levels were higher in yGC than in aGC; in contrast, glycolysis was lower than in aGC. While the *PGAM5* gene silencing, the metabolite levels of glycolysis were higher than those of the siCTL group, and the TCA cycle and pentose phosphate pathway had minimal elevation (Figure 4b). Further testing the changes in metabolic pathways, the important enzymes that regulate glycolysis were all down‐regulated in *siPGAM5* GC. However, the enzymes in the TCA cycle also had a compensatory increased (Figure 4c). Interestingly, the glucose level increased significantly, but the extracellular lactate concentration decreased. We speculate that the decrease in PGAM5 may be the cause of the cellular hyperfusion of the mitochondria, so that the cells are using a lot of mitochondria for energy production and possibly consuming glucose through other metabolic pathways (Figure 4d). Consistently, cellular ATP production was significantly decreased due to aging, but enhanced instead after *PGAM5* knockdown (Figure 4e). Biogenesis is affected in aging cells, and many transcription factors are involved that are key effectors of mitochondrial biogenesis. Publicly available ChIP and sequencing data suggest that CREB1 can bind directly to the *PGAM5* promoter (Figure 4f). ChIP‐PCR with GC showed that the binding affinity of endogenous *PGAM5* in aged GC was significantly higher than that of young GC (Figure 4g).

### 2.5 Imbalance of mitochondrial dynamics in the aged CC from patients

**FIGURE 5:**
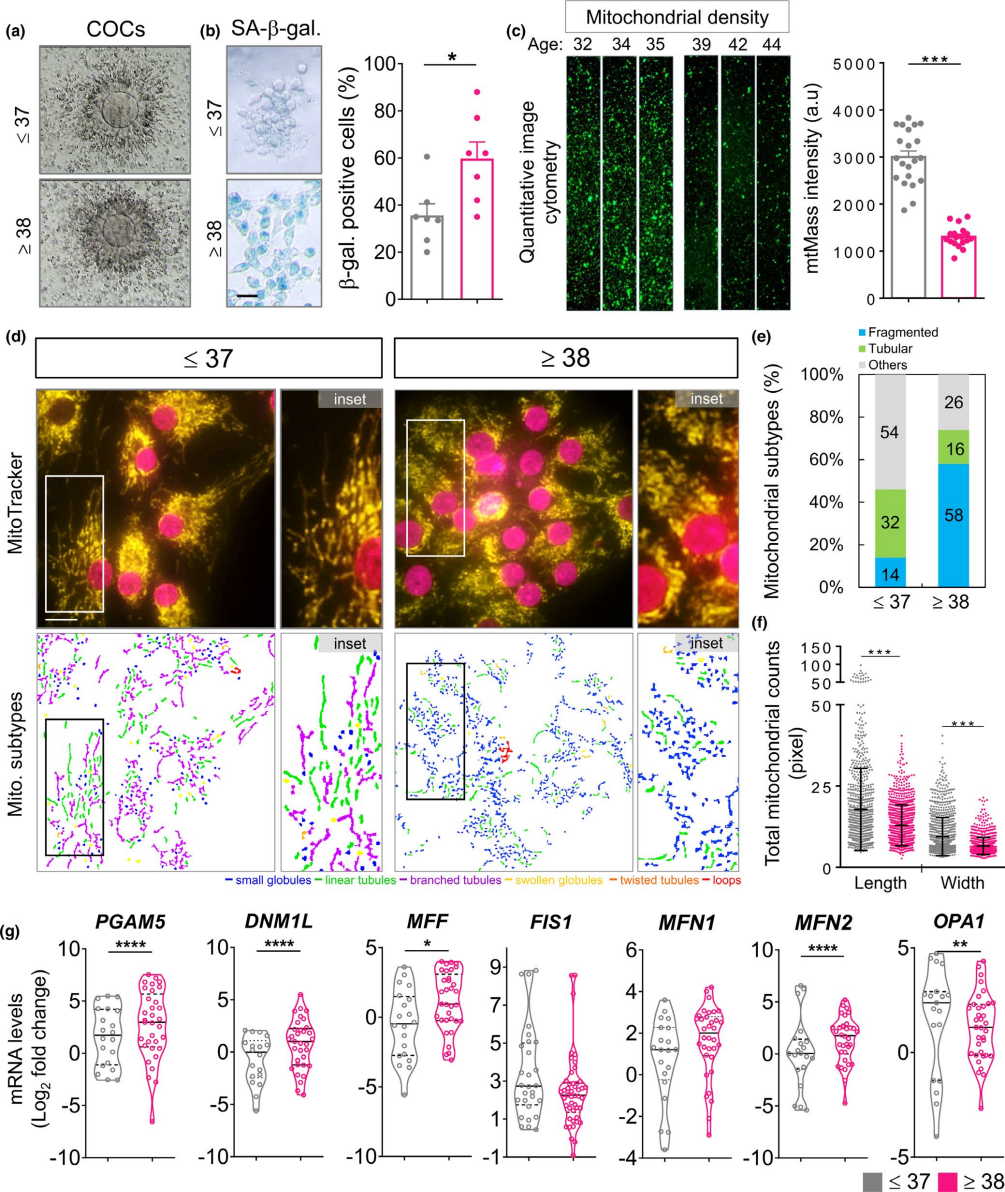
Mitochondrial architecture and gene expression in CC of patients. (a) The representative cumulus–oocyte complexes (COCs) from the ≤37 and ≥38 groups were shown. (b) The representative CC with senescence‐associated β‐galactosidase (SA‐β‐gal) staining from the ≤37 and ≥38 groups were shown. (c) CC were stained with MitoTracker green, and the mitochondrial mass was measured by image cytometry. The relative mean of mitochondrial mass intensity was calculated. (d) CC were stained with MitoTracker and observed by fluorescence microscopy to analyze the mitochondrial network structure. Mitochondria inpatient CC were labeled with MitoTracker and subjected to image capture. The mitochondria were classified according to their morphology: globules, linear tubules, branched tubules, twisted tubules, and loops. (e) Three major types of mitochondria were quantified: fragmented, tubular, and others. (f) The total length and width of each mitochondrion were evaluated. (g) Quantitative qPCR reaction analysis for the ≤37 and ≥38 groups. *p < 0.05, **p < 0.01, ***p < 0.001, and ****p < 0.0001. Scare bar = 20 µm

To validate the clinical relevance of mitochondrial function, the cumulus cells from the patients experiencing infertility were analyzed. The amount of eggs gradually declines in older women from the age of 32, and the decline escalates after the age of 37, reflecting a decline in the egg quality (Baird et al., 2005). Therefore, the patients were divided into two groups, one ≤37‐year group and the other ≥38‐year group. As shown in Figure 5a, the maturation of oocytes and the expansion of the cumulus were higher in the ≤37‐year group. Table 1 shows the baseline features of the ≤37‐year and ≥38‐year groups. A total of 70 women undergoing IVF cycles participated in this study. Basic demographic characteristics, such as body mass index, types of infertility, and basal FSH, E2, LH were not significantly different among the two groups. However, members of the ≤37‐year group were significantly younger than those of the ≥38‐year group (34.2 ± 2.2 years vs. 41.2 ± 2.2 years, *p* < 0.001). Furthermore, the duration of infertility, as well as levels of AMH, was markedly higher in the ≤37‐year group than in the ≥38‐year group. Regarding the duration of stimulation, the HMG/FSH dose, maturation rate, and fertilization, there were no significant differences among the two groups, and there were significant differences in other parameters (Table 2). The senescent phenotypes of the CC were further assessed. Compared with the elderly group, the CC of the ≤37‐year group showed a significant decrease in the percentage of SA‐β‐gal positive cells (Figure 5b). To further evaluate the mitochondrial function of the two groups, the flow cytometric analysis, detecting the mitochondrial mass was used. Using the fluorescent MitoTracker green to visualize mitochondria, the mass of mitochondria observed in CC of the ≥38‐year group was lower than that of the ≤37‐year group (Figure 5c). To assess the dynamics and structure of mitochondria in the ≥38‐year group, the mitochondrial network was analyzed. After visualizing the mitochondria with the fluorescent MitoTracker, the elongation of the mitochondria of ≥38‐year group was significantly found to reduce, and the mitochondria were spherical in shape (Figure 5d). It is worth noting that the mitochondria of CC in the ≤37‐year group have a longer elongation at the ends. Thus, this data showed that the ≤37‐year group had a significantly higher percentage of linear and other mitochondria than that in the ≥38‐year group (Figure 5e). Also, the average length and width of mitochondria in the ≤37‐year group was significantly greater than those in the ≥38‐year group (Figure 5f). To explore the underlying mechanism of the observed reduction in mitochondrial morphology in the ≥38‐year group, the relative expression of mitochondrial dynamic genes was analyzed using the qPCR. The mRNA levels of *PGAM5*, *DNM1L*, and *MFF* in CC of ≤37‐year group were significantly lower than those of ≥38‐year group (Figure 5g). Also, the mRNAs of MFN2 and OPA1 in CC in the ≤37‐year group were higher than those in the CC in the ≥38‐year group. Thus, the biomarkers of programmed cell death, including mitophagy and apoptosis were confirmed. The results showed that PARKIN was significantly reduced in the ≥38‐year group. On the contrary, the levels of the mitochondrial‐regulated intrinsic pathway genes *Cytochrome*‐*c*, *BAX*, *Casp 9*, and *Casp 3* were significantly increased (Figure S1). This result suggested that in the aged CC, mitochondrial fragmentation increases and moves toward the apoptotic pathway, instead of mitophagy to eliminate cells.

**TABLE 1:** Basic characteristics of patients in the young and old groups

Characteristic	≤37 (*n* = 46)	≥ 38 (*n* = 58)	*p* value
Age (years)	34.2 ± 2.2	41.2 ± 2.2	0.0001
Body mass index (kg/m^2^)	22.9 ± 3.7	23.1 ± 3.8	ns
Duration of infertility (years)	3.3 ± 2.1	6.0 ± 4.8	0.0047
Previous IVF failure (*n*)	1.0 ± 1.4	2.6 ± 2.4	ns
Types of infertility (%)			
Primary infertility	25/46 (45.6%)	31/58 (53.4%)	ns
Secondary infertility	21/46 (45.6%)	27/58 (46.5%)	ns
Basal FSH (IU/L)	4.7 ± 2.7	5.7 ± 4.0	ns
Basal LH (pg/ml)	4.0 ± 2.4	5.2 ± 8.1	ns
Basal E2 (IU/L)	101.4 ± 83.4	97.4 ± 78.6	ns
AMH	2.4 ± 2.3	1.4 ± 1.8	0.0496

Note

Values presented as mean ± SD or number (%).

Abbreviations: E2, estradiol; FSH, follicle stimulation hormone; IVF, in vitro fertilization; LH, luteinizing hormone.

**TABLE 2:** Cycle characteristics and pregnancy outcome in the young and old groups

Characteristic	≤37 (*n* = 46)	≥38 (*n* = 58)	*p* value
Stimulation (days)	10.6 ± 1.9	10.7 ± 1.7	ns
HMG/FSH dose (IU)	3022.0 ± 763.8	2951.3 ± 760.6	ns
No. of oocytes retrieved (*n*)	8.3 ± 6.2	4.8 ± 3.6	0.0038
No. of metaphase II oocytes (*n*)	4.8 ± 4.3	2.7 ± 2.7	0.0165
Maturation rate (%)	59.0 ± 26.2	52.4 ± 34.0	ns
No. of fertilized oocytes (*n*)	5.4 ± 3.7	3.5 ± 3.1	0.0197
Fertilization rate (%)	70.9 ± 16.8	71.6 ± 27.3	ns
No. of day 3 embryos (*n*)	4.8 ± 3.1	3.0 ± 2.6	0.0118
No. of top‐quality D3 embryos (*n*)	2.1 ± 2.3	1.0 ± 1.3	0.0153
Clinical pregnancy rate	24/46 (52.1%)	8/58 (13.7%)	0.0016
Ongoing pregnancy rate	22/46 (47.8%)	8/58 (13.7%)	0.0015
Live birth rate	21/46 (45.6%)	8/58 (13.7%)	0.0001

Abbreviations: FSH, follicle stimulation hormone; HMG, human menopausal gonadotrophin.

### 2.6 The clinical significance of PGAM5 in female infertility

**FIGURE 6:**
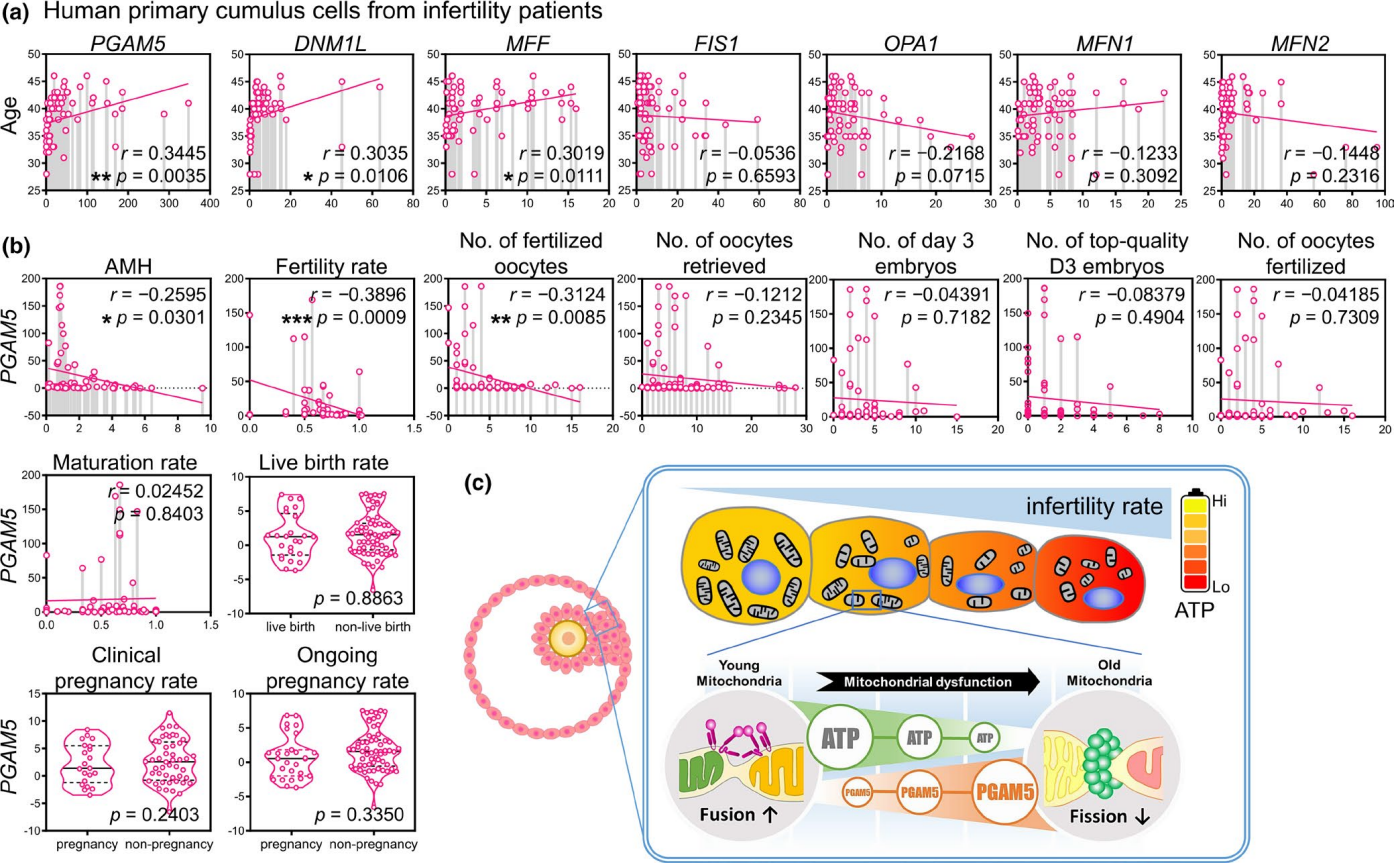
The correlation between PGAM5 expression and mitochondrial dynamics genes cumulus cells of patients undergoing IVF. Scatter plot illustrating the Spearman's correlation of normalized reads per patient between age and PGAM5, as well as mitochondrial dynamics genes in (a). (b) Linear regression analysis of the correlation between the cycle characteristics and the *PGAM5* expression level in patients undergoing IVF Cycles. Spearman's rank correlation coefficients *r* and *p* value were provided in each plot. (c) A working model of the effects of PGAM5 female infertility during reproductive aging. *p < 0.05, and **p < 0.01

To verify the clinical correlation between PGAM5 and age, the correlation between age and each gene was analyzed. We recruited 70 infertility patients and found positive correlations between age and *PGAM5*, *DNM1L*, and *MFF*; there was no significant correlation with the other genes (Figure 6a). The correlation between *PGAM5* and clinical parameters was further analyzed, and the results showed that the AMH, fertility rate, and the number of fertilized oocytes had a significant correlation (Figure 6b). An important limitation of our study was its small sample size. Therefore, an increasing trend was observed in clinical outcomes, but it was not significant. Although there was no significant difference in *PGAM5* expression between pregnant and non‐pregnant in both clinical pregnancy rate and ongoing pregnancy rate. Thus, the data should be interpreted carefully. In summary, these results indicate that PGAM5 plays an important role in the energy capacity of mitochondria in cells and ultimately affects the vulnerability to the damage and development of the female reproductive system in humans.

## 3 DISCUSSION

The complex cumulus–oocyte interaction plays a role in determining energy utilization during the development of mammalian oocytes (Dumesic et al., 2015, 2016). The production of pyruvate in the cell, from glucose, provides the oocytes with the key energy substrate for the mitochondrial oxidative phosphorylation, and the quality of the oocytes also determines the conversion of energy metabolism (Cetica et al., 2002; Dumesic et al., 2015; Seli et al., 2014; Sutton‐McDowall et al., 2010). These metabolic processes rely on the autocrine and paracrine signal transduction, cell junctions, and transzonal processes. With the help of high energy, energy‐rich substrates are transferred from cumulus cells to oocytes. Cumulus cell mitochondria regulate ATP production, purine synthesis, microtubule polymerization, and chromosome segregation (Lin et al., 2017).

The regulation of mitochondrial dynamics is important for the maturation of oocytes. By locally increasing the density of mitochondria, the demand for ATP can be met at a specific location without involving all mitochondria in the cytoplasm, thereby ensuring that the mitochondria are exposed to lower levels of oxidative stress (Brevini et al., 2005; Igarashi et al., 1997). The normal mitochondrial structure is essential for balancing mitochondrial fusion and fission for cell viability, aging, and energy metabolism (Schrepfer & Scorrano, 2016). Previous studies have pointed out that the old CC show a gradual decline in MFN1 and MFN2 expression, associated with mitochondrial fusion (Hou et al., 2019; Picca et al., 2016; Wang et al., 2019). Moreover, the globular mitochondria dominate the primordial follicle and do not require a lot of energy, while the secondary follicles contain a large number of elongated mitochondria, suggesting that the linear mitochondria produce ATP in their mature form (Fair et al., 1997). A previous study has found that the mitochondria of poor ovarian responder patients are donut shaped; the mitochondria of normal ovarian responder are elongated (Li et al., 2018; Lin et al., 2017).

In this study, the mitochondria in GC and CC were studied and found age‐related changes in the mitochondrial ultrastructure. The quality of mitochondria and the MMP of the aged group decreased. However, ROS levels in cells and mitochondria did not decrease significantly in the aged GC. Finally, the decreased ATP production and mitochondrial dynamic imbalance were observed in this study, which may be mainly related to age‐related GC and CC dysfunction. This result indicates that DRP1 recruits PGAM5 to co‐regulate the mitochondrial morphology of aged GC and CC. However, the proportion of PGAM5 in older women is very high, which is also reflected through the production of ATP in the cells of infertile patients, and PGAM5 is correlated with clinical parameters of infertile patients. Not only that, as the women age, the transmission of this energy metabolism also increases significantly, and energy is produced through glycolysis, which explains the decline in the physiological functions of mitochondria in the aged group.

Mitochondria are multifunctional organelles that can store energy and participate in a variety of stress response processes in the cells. It is constantly stimulated by ROS. Excessive ROS can cause protein modification, mtDNA damage, etc., and ultimately lead to mitochondrial dysfunction (Sekine & Ichijo, 2015). PGAM5 is a mitochondrial membrane protein with a molecular size of 26 kD. Previous studies have confirmed that PGAM5 can promote the translocation of BAX to mitochondria and the dephosphorylation of DRP1, and the formation of the BAX/PGAM5/DRP1 complex is necessary for the execution of mitochondrial endogenous apoptosis (Xu et al., 2015). However, PAGM5, DRP1, and BAX exist in the cytoplasm as multimers and are recruited to mitochondria by receptors on the outer mitochondrial membrane, and have the effect of catalyzing the division of mitochondria in the cells (Liu & Chan, 2015; Ma et al., 2020). The lack of PGAM5 is important for regulating aging as it leads to excessive mitochondrial fusion and reduced mitochondrial turnover, which accelerates with aging in mice (Yu et al., 2020). In support, we showed that PGAM5 promoted DRP1 mitochondrial localization by forming complexes with DRP1, leading to increased DRP1‐Ser616 phosphorylation (Figure 4b) in aged GC. However, the KD of PGAM5 significantly inhibited and reduced the DRP1‐mediated mitochondrial breakage caused by aging (Figure 5e–g), but did not change other fission‐related genes, such as FIS1 and MFF. Similar to the results of our study, the aged patients not only have extremely high PGAM5 levels but also have extremely significant differences in the levels of DRP1 and BAX. In turn, the mitophagy of aging cells is reduced, and the level of PGAM5/DRP1/BAX is increased. This result is also reflected in the oocyte quality and conception rate as clinical outcomes.

Lower ATP and reduced mtDNA copy numbers are not only related to poor oocyte quality but also the decline of embryonic development quality, implantation, and placental implantation rate (Van Blerkom et al., 1995; Wai et al., 2010; Wakefield et al., 2011). As the number of mitochondria decreases, the negative effects of poor mitochondrial quality (mutation load) may become more pronounced. The ratio of mutant to wild‐type mitochondria in oocytes may be low enough for normal embryo development, but the increase in this ratio account for the increasing number of offspring diagnosed with mitochondrial diseases (Schaefer et al., 2008). Thus, PGAM5 not only interacts with DRP1 but also is related to the enzymes of anaerobic respiration. Among them, the catalysis of PFKB1 to −4 is the most important regulator of PGAM5‐mediated glycolysis. At the same time, our results confirm that glycolysis supports aging in GC and shows a compensatory increase in the activity of the TCA cycle, promoting the up‐regulation of oxidative phosphorylation of complex I, but itself does not react to the level of ATP. Therefore, the excessive division of mitochondria in the aging germ cells leads to abnormal metabolic function and energy decline. In addition to the interaction between PGAM5 and DRP1 in this study, PGAM5 is negatively correlated with the fertilization rate and the number of fertilized eggs in CC in patients experiencing infertility.

Abnormal mitochondrial dynamics have been reported to be associated with ovarian insufficiency in many studies. PGAM5 activates DRP1 through dephosphorylation and induces mitochondrial fracture and necrosis (Wang et al., 2012; Yu et al., 2020). DRP1 is a key gene in oocyte maturation (Udagawa et al., 2014), and our findings show that PGAM5 is more significantly expressed than DRP1 in older populations, and we hypothesize that PGAM5 is not only associated with mitochondrial dynamic homeostasis but also, most importantly, PGAM5 is directly related to energy metabolism (Figure 4). The most critical factor for oocyte maintenance is the ability of mitochondria to maintain ATP production, and PGAM5 has a dual role in mitochondrial fusion homeostasis and energy metabolism. Therefore, reducing PGAM5 overexpression is a key factor to ensure proper oocyte and mitochondrial activity, which is essential to maintain the physiological function of germ cells to avoid oxidative damage and energy deficiency and, thus, slow down the aging process.

In conclusion, one of the highlights of this study is that the dysfunction and aging of GC and CC are mainly related to impaired mitochondrial function, especially, to the dynamic function of mitochondria. This study confirmed that reducing the excessive division of aging GC and CC and avoiding the production of ATP through glycolysis may restore the fertility of elderly women receiving ART. A small number of CC and GC cultured in vitro in individual patients have a short survival time, which limits further research on the mechanism. This problem can be solved by establishing alternative animal models. The reasons for the decreased mitochondrial division and how to improve it still need further research. Overall, our current study for the first time characterizes PGAM5 as an important mediator in the aging mechanism of germ cells acting through the disruption of mitochondrial homeostasis.

## 4 EXPERIMENTAL PROCEDURES

### 4.1 Cell culture

Human ovarian granulosa cell lines (HGL5) were purchased from Applied Biological Materials (abm) Inc. The cells were maintained in the DMEM/F12K medium containing 10% FBS, 1% Penicillin/Streptomycin, 2% Ultroser G (Pall Corp.), and 1% ITS plus (Zen‐Bio). Early passage cells (<P10) were considered young HGL5 GC, and late passage cells (>P50) were considered aged HGL5 GC. The cells were maintained at 37°C in a humidified atmosphere with 5% CO_2_.

### 4.2 Senescence‐associated β‐galactosidase staining

The senescence‐associated β‐galactosidase (SA‐β‐gal) staining was performed as described (Lin et al., 2017).

### 4.3 The telomerase activity assay

The quantitative determination of telomerase activity was performed using the Telomerase activity quantification qPCR assay kit (Sciencell Research Lab., Inc.) according to the manufacturer's protocol.

### 4.4 The mitochondrial functional analysis

The mitochondrial function assays were performed as previously described (Tsui et al., 2019). The cells were collected, washed, re‐suspended in the culture medium, and stained with the DCFDA (5 μM), MitoTracker green (10 nM), MitoSOX (5 μM), (Molecular Probes), and ATP (BioTracker ATP‐Red Live Cell Dye, Merck) at 37°C for 30 min. The cells were washed twice with PBS, trypsinized, and collected, and the pellet was re‐suspended in PBS for analysis using the flow cytometry.

### 4.5 The reverse transcriptase‐polymerase chain reaction (qPCR) and PCR array

The total RNA was extracted with the EasyPrep Total RNA Kit (Biotools). A total of 1 μg of RNA was reverse‐transcribed with a ToolScript MMLV RT kit (Biotools) for cDNA synthesis. Real‐time polymerase chain reaction (qPCR) was carried out using a StepOnePlusTM system (Applied Biosystems) with TOOLS 2× SYBR qPCR Mix (Biotools). The expression levels of all the genes in cells were normalized to the internal control RNU6‐1 gene. All the samples with a coefficient of variation for *C*_t_ value >1% were retested. RT2 Profiler custom PCR array (Qiagen) was used to check the mRNA levels of 84 genes. GAPDH mRNA expression was detected as a reference gene. Negative control was included in each run. The total RNA was extracted with REzol (Protech Technology). The mRNA expression level was analyzed using the SYBR Green‐based quantitative real‐time PCR (qRT‐PCR) analysis (StepOne; Applied Biosystems), with β‐actin and RNU6‐1 as the reference genes. Refer to the Table S1 for related primer sequences.

### 4.6 Immunoprecipitation

Immunoprecipitation was performed with the total lysate of cells using a Dynabeads Protein G Kit (Novex, ThermoFisher Scientific). Briefly, 5 μg primary antibody (Table S2) was incubated with Dynabeads for 10 min at RT, and the resulting mixture was incubated with 500 μg total protein lysate for 10 min at RT. The beads were washed with PBST (phosphate‐buffered saline containing 0.02% Tween‐20) and heated to 70°C for 10 min. The tube was then placed on a magnet, and the supernatant was loaded onto a gel.

### 4.7 Immunoprecipitation and immunoblotting

Immunoprecipitation was performed in the total lysate of GC using a dynabeads protein G kit (Novex, ThermoFisher Scientific). Briefly, the primary antibody was incubated with dynabeads for 10 min at room temperature (RT), and then incubated with total protein lysate for 10 min at RT. After washing with PBST buffer (0.02% Tween‐20) and heating with 70°C for 10 min, the tube was placed on the magnet and the supernatant was loaded onto a gel. For immunoblotting, the total proteins were subjected to SDS/PAGE, transferred to PVDF membranes, and probed with primary antibodies. Immunoreactive detection was performed using a chemiluminescence detection system (GE Healthcare).

### 4.8 Immunofluorescence

The cells or tissue were fixed with 4% paraformaldehyde for 10 min, permeabilized with 0.2% Triton X‐100 for 5 min, blocked with 3% BSA for 0.5 h, and incubated with the indicated first antibodies (Table S2) overnight at 4°C. After washing with PBST, the samples were incubated with the secondary antibodies for 0.5 h at RT. All glass slides were digitized with BX61VS^®^ Fully Motorized Fluorescence Microscope (Olympus Corporation) at ×20 (0.26 μm/pixel) with High precision (High precision autofocus). BX61VS whole‐slide images were viewed and analyzed with Olympus VS‐ASW^®^ software at Li‐Tzung Pathology Laboratory (Kaohsiung, Taiwan).

### 4.9 siRNA transfections

For siRNA transfection, HGL5 cells were transfected with 200 pmole of indicated siRNA using Lipofectamine 3000, and analyzed 48 h after transfection. siRNA sequences used in the study were as follows: PGAM5 siRNA (ID: s46937) and negative control siRNA (4390843; ThermoFisher Scientific).

### 4.10 Animals

Young BALB/c mice (<10 weeks old) and aged female mice (>44 weeks old) were purchased from National Laboratory Animal Center. Mice were maintained in cages at 25°C ± 2°C on a 12‐h light/dark cycle, with free access to food and water. All animal studies were handled in accordance with procedures approved by the Institutional Animal Care and Use Committee of Kaohsiung Veterans General Hospital (#2020‐2021‐A020).

### 4.11 Patients and design

CC were obtained from 70 women experiencing infertility and undergoing IVF in the ART Center of the Kaohsiung Veterans General Hospital from January 2016 to March 2020. The infertile patients who underwent IVF cycles were enrolled and divided into two groups: (i) younger group (patients aged ≤37 years); (ii) older group (patients aged ≥38 years who underwent IVF cycles). The exclusion criteria included: (i) patients who underwent oophorectomy; (ii) patients exposed to cytotoxic or pelvic irradiation for malignancy; and (iii) patients who had undergone treatment with herbal drugs, nutritional supplements, or other hormonal agents. The basic characteristics of patients and cycle variables, including the numbers of retrieved oocytes, metaphase II oocytes, fertilized oocytes, top‐quality day‐3 embryos, and transferred embryos were analyzed among the younger and older groups.

### 4.12 Collection of human CC

The patient's COCs were collected after oocyte aspiration, washed, and incubated in the IVF medium covered with paraffin oil. The cumulus cells were exposed to 40 IU/ml hyaluronidase (SynVitro™ Hyadase, Origo) for 3 min before being washed with PBS. After removing the oocytes, the cumulus cells were mechanically broken down and washed. The CC pellet was re‐suspended in Histopaque 1077 (Sigma), supplemented with 10% FBS (Gibco, Thermo Fisher Scientific), 5 mg/L insulin, 5 mg/L transferrin and 5 μg/L sodium selenite (ITS, Sigma), and 1.25 μM Androstenedione (4‐androstene‐3, 17‐dione, Sigma). The CC were plated in a four‐well plate at a concentration of 2 × 10^4^ viable cells/well to be cultured at 37.5°C in a humidified incubator at 5% CO_2_ for up to 24 h for further study.

### 4.13 Ethics statement

This study was approved by the institutional review board of the Kaohsiung Veterans General Hospital and was performed according to approved guidelines (VGHKS14‐CT10‐16, VGHKS19‐CT6‐17). All participants signed informed consent. This study was implemented in compliance with the Declaration of Helsinki. This study was registered with the Clinical Trial Registry (Identifier: NCT03438812).

### 4.14 The multi‐omics analysis tool

Metascape is a free gene annotation and analysis resource that combines functional enhancements, interactive genome analysis, gene annotation, and membership search to take advantage of the knowledge base in an integrated portal (Zhou et al., 2019). The GeneMANIA database contains information from many sources, including experimental data, computational prediction methods, and public text collections. This resource also uses many functional classification systems to highlight the richness of functions in the protein list provided by the user (Szklarczyk et al., 2019).

### 4.15 UHPLC‐MS/MS analysis

The cells were collected for metabolite analysis by NTU Centers of genomics and precision medicine MetaCore. The detailed materials and methods are based on the previously published (Liao et al., 2017).

### 4.16 Statistical analysis

Each experiment was performed at least three times, and all data are represented as mean ± standard error of the mean (SEM) of the quadruplicate measurements. The intensity of fluorescence was quantified and analyzed using the ImageJ software (NIH), Zen lite software (Carl Zeiss Co. Ltd.), and MicroP software, respectively (Peng et al., 2011). The statistical significance was evaluated using a two‐way analysis of variance (ANOVA) test using GraphPad Prism 8.0 (GraphPad Software). The differences were considered significant when *p* < 0.05.

## CONFLICT OF INTEREST

The authors disclose no potential conflicts of interest.

## AUTHOR CONTRIBUTIONS

CJ Li and KH Tsui prepared the manuscript. CJ Li and LT Lin performed the experiments and analyzed the data. HW Tsai and ZH Wen participated in discussions and provided critical intellectual input. CJ Li and KH Tsui conceptually designed the strategy used for this study, interpreted the data, provided intellectual input, supervised the studies, and wrote the manuscript.

### OPEN RESEARCH BADGES

This article has earned an Open Materials, for making publicly available the digitally‐shareable data necessary to reproduce the reported results. The data is available at https://doi.org/10.3390/diagnostics10050295.

## Supporting information

Supplementary Material

## Data Availability

Data sharing is not applicable to this article, as no new data were created or analyzed in this study.
