# Supplementary material for: Phosphoglycerate mutase family member 5 maintains oocyte quality via mitochondrial dynamic rearrangement during aging
Source: Aging Cell. 2022 Jan 7;21(2):e13546. doi: 10.1111/acel.13546 (PMC8844125; doi:10.1111/acel.13546)
Supplement: Supplementary file 1 — Supplementary Material [file ACEL-21-e13546-s001.docx]

Supplementary Table 1.Primer sequences designed for RT-PCR

| **Primers** | Forward | Reverse |
| --- | --- | --- |
| MFN1 | F-GAGGTGCTATCTCGGAGACAC | R-GCCAATCCCACTAGGGAGAAC |
| MFN2 | F-CACATGGAGCGTTGTACCAG | R-TTGAGCACCTCCTTAGCAGAC |
| OPA1 | F-TGTGAGGTCTGCCAGTCTTTA | R-TGTCCTTAATTGGGGTCGTTG |
| DRP1 | F-ACCCGGAGACCTCTCATTCT | R-TGACAACGTTGGGTGAAAAA |
| FIS1 | F-GATGACATCCGTAAAGGCATCG | R-AGAAGACGTAATCCCGCTGTT |
| DNM1L | F-GATGCCATAGTTGAAGTGGTGAC | R-CCACAAGCATCAGCA AAGTCTGG |
| PGAM5 | F-CAA ACC CAG AAT TGT TCT CCT T | R-ATG TGG TCT TCC TGA ATC CCT |
| PINK1 | F-CAA GCA AGTGTC TGA CCC AC | R-GCT TCA TAC ACA GCG GCA TT |
| PARKIN | F-ACCCACCTACCA CAG CTT TT | R-CAA GGT GAG GGT TGC TTG TC |
| Cyto. c | F-TTTGTTGGGCAGTCCTGATT | R-GATGGCACTCACCATCTTTG |
| BCL2 | F-CTGGTGGGAGCTTGCATCAC | R-ACAGCCTGCAGCTTTGTTTC |
| BAX | F-GCTGTTGGGCTGGATCCAAG | R-TCAGCCCATCTTCTTCCAGA |
| Casp9 | F-AGGGAGTCAGGCTCTTCCTT | R-ATGTCCACTGGTCTGGGTGT |
| Casp3 | F-ATTGTGGAATTGATGCGTGA | R-GGCAGGCCTGAATAATGAAA |
| HK2 | F-TCCGTAACATTCTCATCGATTTCA | R-TGTCTTGAGCCGCTCTGAGAT |
| GPI | F-GACCCAGCACCCCATACG | R-CAAGAAGTTGGCCAGGAGGAT |
| ENO1 | F-TGGGAAAGCTGGCTACACTGA | R-CTCGGAGGCCGCTACGT |
| ENO2 | F-AAGGCTGGCTACACGGAAAA | R-CGATAAAACTCTGAGGCAGCAA |
| PKM | F-TCTGAGCGGTCTTTGCTAGTGA | R-TGACATAATGCTCCCCTTTTGG |
| LDHA | F-GAAGCGGTTGCAATCTGGAT | R-GGTGAACTCCCAGCCTTTCC |
| LDHB | F-GGGAACATGGCGACTCAAGT | R-GAGAAACACCTGCCACATTCAC |
| LDHC | F-GGGCTATTGGACTGTCTGTGATG | R-TGGGTGCACTCTCCTAAGATTTTT |
| PDHA1 | F-ACCCCACAGACCATCTCATCA | R-CCCCGGGTGAAAGTAAAGC |
| PDHB | F-AACTGTGGTTTCCCATTCAAGAC | R-TTAGATAGCACTGCTGCAGCTTCT |
| CS-F | F-TCTGGAGCCGAGCCTTAGG | R-GACCCTCTGTGCTCATGGACTT |
| MDH1 | F-GCTGTCATCAAGGCTCGAAAAC | R-GGTCACAGATGGCTTTTGCA |
| MDH2 | F-TGCCCGGAAGCCATGAT | R-GACTCGAGCTGGATCCAAACC |
| SUCLA2 | F-GCA AGA AGCTGG TGT CTC CGT T | R-GGC AAC ACC AAG CTT TGC A |
| KGDH | F-TGC TCG GCA ATT CAG TCA TC | R-GCC AGT GTG CCA TCG CTT A |
| IDH1 | F-CGG AAC CCA AAA GGT GAC AT | R-TGG CAA CAC CAC CAC CTT CT |
| IDH2 | F-CCT GGC GGG CTG CAT | R-GGA AGT GCT CGT TCA GCT TCA |
| ACO1 | F-GGT TTG ACG TGG TGG GCT AT | R-TCA GGT AAA GGC CCA CTG TTG |
| ACO2 | F-TCA ACC CAG AGA CCG ACT ACC T | R-GAG CCT CCA GCC TGA ACT TCT |
| P16 | F-TGTGTTGGAGTTTTCTGGAGTGA | R-CAAGAAATGCCCACATGAATGT |
| P21 | F-TGGAGACTCTCAGGGTCGAAAA | R-GCGTTTGGAGTGGTAGAAATCTG |
| P27 | F-AGC GCA AGT GGA ATT TCG A | R-GCC ACT CGT ACT TGC CCT CTA |
| RNU6-1 | F-CTCGCTTCGGCAGCACATATACT | R-ACGCTTCACGAATTTGCGTGT C |

Supplementary Table 2. 1^st^ antibodies for immunoblotting, immunofluorescence and IP

| **Antigen** | **Host** | **Cat.** | **Type** | **Source** |
| --- | --- | --- | --- | --- |
| DRP1 | Mouse | ab56788 | monoclonal | Abcam |
| DRP1 Ser616 | Rabbit | 3455 | monoclonal | Cellsignailing |
| FIS1 | Rabbit | GTX111010 | polyclonal | Genetex |
| MFN1 | Rabbit | GTX133351 | polyclonal | Genetex |
| MFN2 | Mouse | ab56889 | monoclonal | Abcam |
| OPA1 | Rabbit | GTX129917 | polyclonal | Genetex |
| PGAM5 | Rabbit | ab126534 | polyclonal | Abcam |
| Total OXPHOS | Mouse | ab110411 | monoclonal | Abcam |
| Beta actin | Rabbit | GTX109639 | polyclonal | Genetex |
